# Identification of Hub Prognosis-Associated Oxidative Stress Genes in Pancreatic Cancer Using Integrated Bioinformatics Analysis

**DOI:** 10.3389/fgene.2020.595361

**Published:** 2020-12-08

**Authors:** Xin Qiu, Qin-Han Hou, Qiu-Yue Shi, Hai-Xing Jiang, Shan-Yu Qin

**Affiliations:** ^1^Department of Gastroenterology, The First Affiliated Hospital of Guangxi Medical University, Nanning, China; ^2^Department of Neurosurgery, Affiliated Tumor Hospital of Guangxi Medical University, Nanning, China

**Keywords:** pancreatic cancer, oxidative stress, prognosis, integrated bioinformatics analysis, risk model

## Abstract

**Background:**

Intratumoral oxidative stress (OS) has been associated with the progression of various tumors. However, OS has not been considered a candidate therapeutic target for pancreatic cancer (PC) owing to the lack of validated biomarkers.

**Methods:**

We compared gene expression profiles of PC samples and the transcriptome data of normal pancreas tissues from The Cancer Genome Atlas (TCGA) and Genome Tissue Expression (GTEx) databases to identify differentially expressed OS genes in PC. PC patients’ gene profile from the Gene Expression Omnibus (GEO) database was used as a validation cohort.

**Results:**

A total of 148 differentially expressed OS-related genes in PC were used to construct a protein-protein interaction network. Univariate Cox regression analysis, least absolute shrinkage, selection operator analysis revealed seven hub prognosis-associated OS genes that served to construct a prognostic risk model. Based on integrated bioinformatics analyses, our prognostic model, whose diagnostic accuracy was validated in both cohorts, reliably predicted the overall survival of patients with PC and cancer progression. Further analysis revealed significant associations between seven hub gene expression levels and patient outcomes, which were validated at the protein level using the Human Protein Atlas database. A nomogram based on the expression of these seven hub genes exhibited prognostic value in PC.

**Conclusion:**

Our study provides novel insights into PC pathogenesis and provides new genetic markers for prognosis prediction and clinical treatment personalization for PC patients.

## Introduction

Pancreatic cancer (PC) is one of the most common tumors worldwide and is a severe threat to human health (Kamisawa et al., 2016). The 5-year overall survival rate of patients with PC is estimated at only 2–9% (Ilic and Ilic, 2016), and by 2030, PC is expected to become the second leading cause of cancer-associated death after lung cancer, ranking above breast and colorectal cancers (Rahib et al., 2014). The poor outcomes of patients with PC are mainly associated with early metastasis, rapid progression, and a lack of sensitive screening tools for early diagnosis (Singhi et al., 2019). To date, surgical resection of cancer tissues remains the most common choice for PC treatment, which effectively increases patients’ 5-year overall survival rate to 20–30%; however, less than 20% of PC patients are eligible for surgical treatment because of advanced-stage diagnoses, at which point cancer has already metastasized (Kamisawa et al., 2016).

In recent years, new developments in targeted molecular therapy, immunotherapy, and neoadjuvant therapy have demonstrated certain beneficial effects for PC; however, several side effects and questionable curative benefits for individual treatment must be addressed (Wu et al., 2019). Therefore, many studies have focused on constructing more effective prediction models that could better clarify the factors contributing to the prognosis and progression of PC, aiming to provide more evidence for individual treatment strategies. Despite these efforts, few screening biomarkers and tools have shown sufficient significance for widespread clinical application in PC. Thus, it is necessary to uncover additional biomarkers and construct novel tools with validated diagnostic value predicting individual diagnosis and prognosis in PC cases.

Oxidative stress (OS) is a pathological phenomenon in which an imbalances between oxidants and antioxidants production that results in the production of high levels of reactive oxygen species (ROS), which represent a potentially critical factor driving tumorigenesis and cancer progression (Brown and Wilson, 2004; Zhou et al., 2017; Kangari et al., 2018). ROS include several reactive non-radical and free radical species, such as singlet oxygen, hydrogen peroxide, and superoxide anion (Lü et al., 2010), which are dramatically elevated in patients with PC (Martinez-Useros et al., 2017). Previous studies have shown that as the scavenging potential is reduced, excessive ROS could damage the DNA causing genotoxicity (Zhou et al., 2013; Wang et al., 2017), eventually inducing genomic mutations that may initiate tumorigenesis (Oates and Gilkeson, 2006; Smith et al., 2010). In PC, ROS are linked to different factors, such as high alcohol intake, cigarette smoking, obesity, and inflammatory conditions (Nöthlings et al., 2005). ROS accumulation can significantly suppress apoptosis in PC cells and contributes to PC tumorigenesis and progression (Vaquero et al., 2004; Yu and Kim, 2014; Martinez-Useros et al., 2017). Accordingly, some compounds targeting OS, such as vitamins (Monti et al., 2012; Patacsil et al., 2012), curcumin (Dhillon et al., 2008; Bimonte et al., 2016), and coenzyme Q10 (Hertz and Lister, 2009) have been proposed as novel chemotherapeutic treatments for PC. Together, the studies discussed above indicate that OS is closely associated with PC progression. Nevertheless, the value of OS-related genes in PC prognosis prediction remains largely unclear, and the underlying mechanisms require further validation.

With the recent development of genomic technologies, bioinformatics analysis has been widely employed for identifying the interaction between gene signatures and tumors (Haqq et al., 2005; Qiu et al., 2015); however, a few studies have focused on identifying gene expression signatures to construct predictive models for patients with PC. Moreover, no systematic study has aimed to discover specific OS-related hub genes that correlate with cancer prognosis or progression. In the present study, we aimed to identify candidate OS genes that are significantly differentially expressed between PC and normal pancreatic tissues based on publicly available data obtained from The Cancer Genome Atlas (TCGA) and Genome Tissue Expression (GTEx) databases. Subsequently, protein-protein interaction (PPI) network construction, univariate Cox regression analysis, least absolute shrinkage and selection operator (LASSO) analyses were performed to identify hub genes among differentially expressed OS-related genes (DEOGs) that were significantly related to PC prognosis. Furthermore, we constructed a prognostic risk model based on hub gene expression and systematically explored each gene function and clinical significance in patients with PC. To the best of our knowledge, this is the first OS-associated risk model for prognostic prediction, which might provide novel insight into PC pathogenesis to tailor personalized treatment and improve the outcome for PC patients.

## Materials and Methods

### Raw Data Acquisition

RNA-sequencing data of 178 PC samples and four normal tissues with corresponding clinical information were acquired from TCGA^1^ (Liu et al., 2019). In addition, the transcriptome data of 167 whole normal pancreatic tissue samples were retrieved from the Genome Tissue Expression (GTEx) database^2^ (Gentles et al., 2015; The GTEx Consortium, 2015). Gene expression profiles and clinical information of patients with PC from the Gene Expression Omnibus (GEO) GSE28735 (including 45 matching pairs of pancreatic tumor and adjacent non-tumor tissues) and GSE62452 (including 69 pancreatic tumor and 61 adjacent non-tumor tissues) cohorts^3^ were downloaded and merged as validation group (Huang et al., 2020). Detailed characteristics of the datasets are listed in Supplementary Table 1. The averages expression values of the probe sets were calculated for the same gene with multiple probe sets (Li et al., 2014). OS genes detected in over 80% of samples were identified, and the minimum non-zero value replaced zero-values in the corresponding gene in the expression matrix (Yan et al., 2019).

To screen out OS-associated genes, 1399 protein domains of OS, with a relevance score ≥ 7 (approximately top 10% OS-related genes), were acquired from the GeneCards database^4^ and subsequently applied for further exploration.

### Differential Gene Expression Analysis

To avoid inaccurate differential expression analysis caused by the small sample size of normal tissues, DEOGs between PC and normal pancreas tissues were identified from the TCGA and GTEx database. Original gene expression data were measured as fragments per kilobase of transcript per million mapped reads (FPKM) and log2-transformed. Furthermore, the RNA expression profiles were normalized with the R package “sva” to remove batch effects, as previously reported (Xiao et al., 2020; Zhang et al., 2020b). Then, the “limma” package in R was applied, and genes with an average count value lower than 1 were all excluded from further analyses. OS-related genes with a false discovery rate (FDR) < 0.05 and | log2 fold change (FC)| ≥ 2, which was calculated utilizing gene expression levels, were regarded as DEOGs in accordance with previously reported methods (Li et al., 2020) and visualized as a volcano plot and heatmap using the “ggplot2” and “pheatmap” packages in R (Wickham, 2009).

### Gene Ontology (GO) and Kyoto Encyclopedia of Genes and Genomes (KEGG) Pathway Enrichment Analyses

Gene ontology and KEGG enrichment analyses of the identified DEOGs were performed to systematically understand the biological functions of the selected OS genes (Pathan et al., 2017). All analyses were performed using the Database for Annotation, Visualization, and Integrated Discovery (DAVID) 6.8 tool (Huang et al., 2009). Genes associated with GO terms and KEGG pathways with P and FDR values < 0.05 were considered to indicate significant enrichment.

### Construction of the PPI Network and Screening of Key Modules

The STRING platform^5^ (Szklarczyk et al., 2019) was used to obtain PPI information for the DEOGs, and then explore the functional interactions between proteins (Szklarczyk et al., 2015). Subsequently, the interaction data were submitted to the Cytoscape 3.7.0 software to construct a PPI network. The Molecular Complex Detection (MCODE) plug-in was used to select the virtual modules and hub genes in the PPI network, with an MCODE score and node count > 5 and *P* < 0.05 (Bader and Hogue, 2003).

### Prognostic Model Construction and Efficacy Evaluation

To identify the prognosis-associated OS genes, hub genes identified in the PPI network were subjected to univariate Cox regression analysis using the “survival” package in R to identify genes that are highly crucial for patients’ survival (Zhang et al., 2020a), with a cut-off criterion of *P* < 0.05. After that, genes identified to be significantly associated with the overall survival of PC patients through the univariate Cox regression analysis were integrated for analysis using LASSO, a widely used machine-learning algorithm, which can preserve valuable variables and avoid overfitting (Jiang et al., 2018), to complete the shrinkage of prognostic OS genes and categorizes patients into high- or low-risk subgroups. In the regression analysis, the normalized gene expression profile of candidate prognosis-associated DEOGs was set as the independent variable, whereas the response variables were the status and overall survival of PC patients. The optimal penalty parameter (λ) was identified via the minimum criteria (i.e., the value of λ was accompanied with the lowest partial likelihood deviance), and 1000 iterations and ten-fold cross-validation was also applied to reduce the coefficient instability. The risk score for each sample was calculated using the following formula:

$$r\,i\,s\,k\,s\,c\,o\,r\,e=\sum_{i=1}^{n}\left(E\,x\,p_{i}^{*}\,\beta_{i}\right)$$

where Exp_*i*_ represents the relative expression value of the *i*^*th*^ OS gene, and β represents the regression coefficient. Genes screened through the LASSO analysis were selected as hub OS genes.

Based on the median risk score, PC patients in the TCGA cohort were stratified into low- and high-risk subgroups. The Kaplan-Meier method and log-rank test using the Kaplan-Meier “survival” package in R were further used to compare survival between two risk subgroups in PC samples (Klein and Moeschberger, 1997). The R packages “survivalROC” and “timeROC” were also applied to validate the predictive accuracy of the gene signature (Heagerty and Zheng, 2005). Univariate and multivariate Cox regression analyses were conducted to evaluate the relationship between clinical characteristics and risk scores. Besides, the same formula and regression coefficients described above were applied to the GSE28735 and GSE62452 validation cohorts to confirm the predictive applicability of our OS-related hub gene prognostic PC signature. Patients in the validation set were also stratified into low- and high-risk groups by the same median risk score calculated from the TCGA database.

### Hub Gene Evaluation

To validate the differential expression of the hub OS genes at the protein level, data from the Human Protein Atlas (HPA) online database^6^ were used to compare the protein levels between normal pancreas and PC tumor tissues (Thul et al., 2017). The expression profile of these OS genes in PC was also verified in TCGA and validation cohorts. Furthermore, the Kaplan-Meier method was applied to estimate each gene’s prognostic value in the TCGA-PC cohort. Finally, a nomogram incorporated with calibration plots was constructed based on the expression of hub prognosis-associated OS genes to be used as a predictive tool for the clinical outcome of patients with PC using the “rms” package in R (Gu et al., 2020).

## Results

### Identification of DEOGs

**FIGURE 1:**
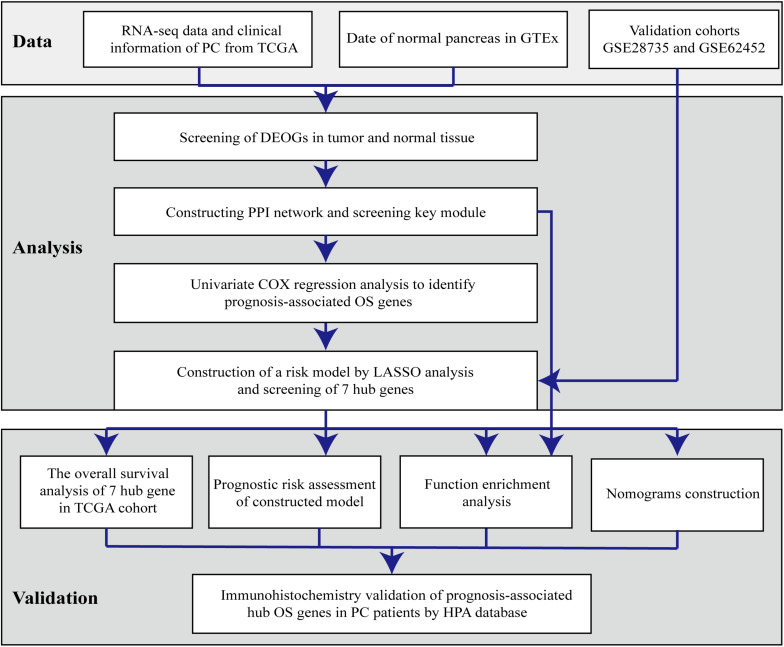
Flowchart describing the schematic overview of the study design.

**FIGURE 2:**
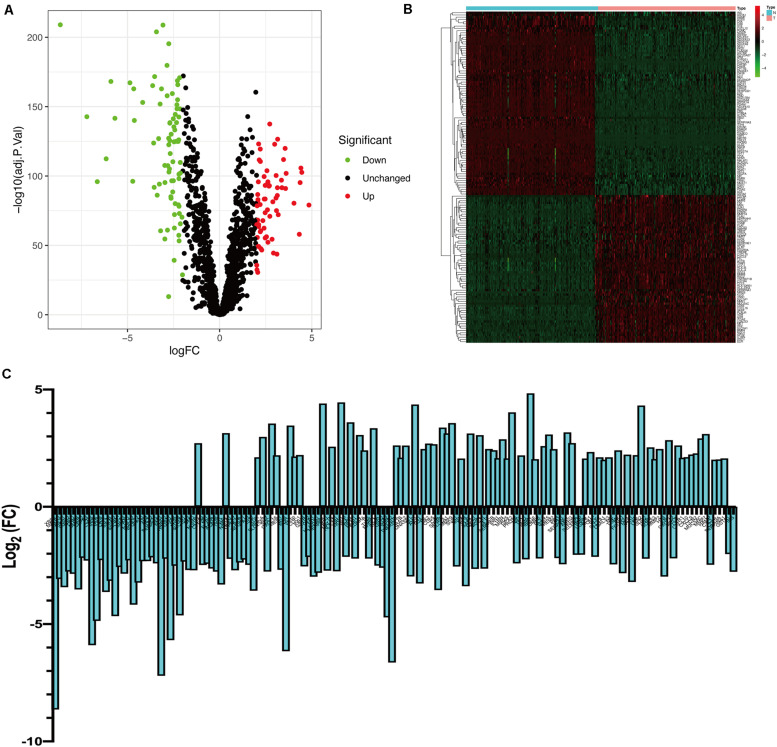
Identification of differently expressed OS genes. **(A)** Volcano plot of DEOGs between TCGA-PC and GTEx-pancreas cohorts. **(B)** Heatmap of DEOGs. **(C)** Histogram of DEOGs.

Bioinformatics analysis of publicly available datasets was performed according to the workflow shown in Figure 1. A total of 1399 OS genes were obtained from the GeneCards database, and their differential expression between PC samples and normal tissues was explored. Of these, 148 genes were screened out as DEOGs in PC (FDR < 0.05 and | log_2_ FC| ≥ 2), including 66 upregulated and 82 downregulated genes. The distribution of these genes is shown in Figures 2A–C.

### Functional Enrichment Analysis of DEOGs

**FIGURE 3:**
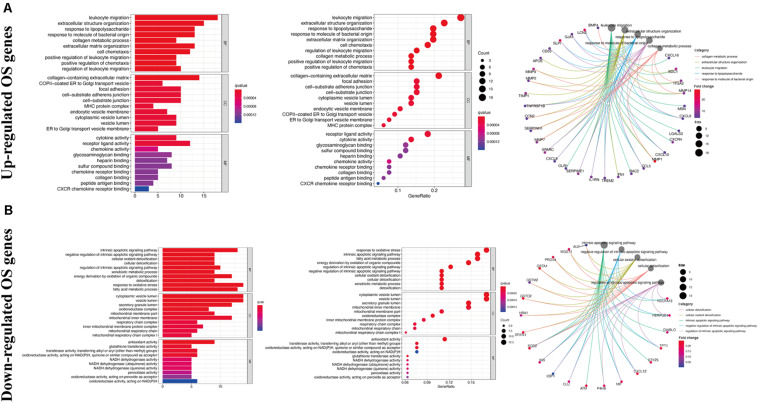
Gene ontology enrichment analysis of DEOGs. **(A)** Top 10 classes of GO enrichment terms about up-regulated DEOGs in biological process (BP), cellular component (CC), and molecular function (MF). **(B)** Top 10 classes of GO enrichment terms about down-regulated DEOGs in BP, CC, and MF.

**FIGURE 4:**
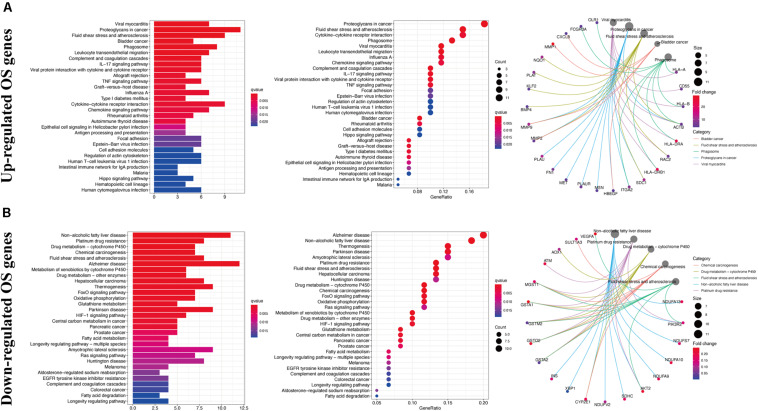
Kyoto encyclopedia of genes and genomes enrichment analysis of DEOGs. **(A)** Top 30 classes of KEGG enrichment terms about up-regulated DEOGs. **(B)** Top 30 classes of KEGG enrichment terms about down-regulated DEOGs.

Gene ontology analysis showed that, with respect to the upregulated DEOGs, the most enriched biological processes included the response to lipopolysaccharide, leukocyte migration, and extracellular structure organization (Figure 3A), whereas relative to the downregulated DEGs, intrinsic apoptotic signaling pathway, cellular oxidant detoxification, and cellular detoxification were most enriched terms (Figure 3B). In terms of cellular components, the upregulated genes were linked to enriched terms such as collagen-containing extracellular matrix, COPII-coated endoplasmic reticulum to Golgi transport vesicle, and focal adhesion (Figure 3A), whereas downregulated genes were associated with cytoplasmic vesicle lumen, vesicle lumen, and secretory granule lumen (Figure 3B). With regard to the molecular function GO terms, upregulated OS genes were linked to enriched terms including cytokine activity, receptor-ligand activity, and chemokine activity (Figure 3A), whereas the downregulated OS genes were associated with glutathione transferase activity, antioxidant activity, and transferase activity (Figure 3B). KEGG pathway analysis showed that the upregulated genes were enriched in viral myocarditis, proteoglycans in cancer, and fluid shear stress and atherosclerosis (Figure 4A), whereas the downregulated genes were mainly enriched in non-alcoholic fatty liver disease, platinum drug resistance, and drug metabolism-cytochrome P450 pathways (Figure 4B).

### Construction of the PPI Network for DEOGs and Screening of Key Modules

**FIGURE 5:**
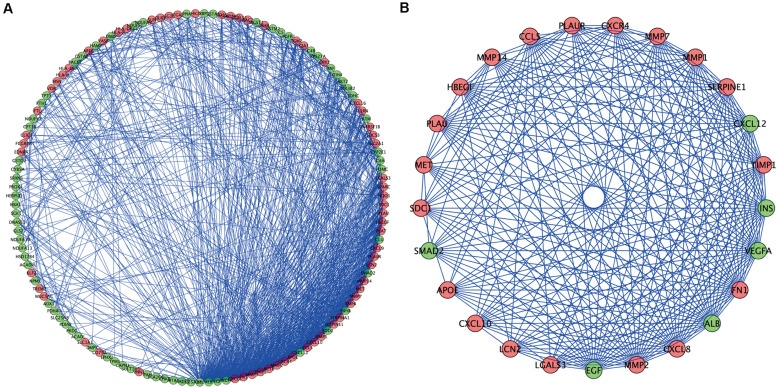
Protein–protein interaction network and modules screening. **(A)** PPI network of DEOGs. **(B)** Critical module from PPI network. Green circles represent down-regulated genes, and red circles represent up-regulated genes.

To further understand the inter-relationship among the DEOGs, we constructed a PPI network with 131 nodes and 934 edges (Figure 5A); in this network, the most significant module was identified to have 25 nodes and 235 edges (Figure 5B). Functional enrichment analysis indicated that the genes in the key module were mainly enriched in leukocyte migration, positive chemotaxis, and cell chemotaxis, whereas KEGG analysis indicated that these genes were significantly enriched in pathways associated with bladder cancer, proteoglycans in cancer, and AGE-RAGE signaling pathway in diabetic complications (Table 1).

**TABLE 1:** Kyoto encyclopedia of genes and genomes pathway and GO enrichment analysis of OS genes in key module.

	**Terms**	***P*-value**	**FDR**
**GO enrichment**			
**Biological processes**	Leukocyte migration	7.68E-10	3.68E-10
	Positive chemotaxis	1.67E-09	7.98E-10
	Cell chemotaxis	1.67E-09	7.98E-10
	Positive regulation of leukocyte migration	1.67E-09	7.98E-10
	Positive regulation of chemotaxis	2.05E-09	9.83E-10
**Cellular component**	Cytoplasmic vesicle lumen	5.43E-09	2.82E-09
	Vesicle lumen	5.43E-09	2.82E-09
	Platelet alpha granule lumen	5.43E-09	2.82E-09
	Platelet alpha granule	2.67E-08	1.38E-08
	Secretory granule lumen	6.08E-08	3.16E-08
**Molecular function**	Receptor ligand activity	6.77E-08	3.08E-08
	Heparin binding	7.72E-06	3.51E-06
	Chemoattractant activity	1.08E-05	4.89E-06
	CXCR chemokine receptor binding	1.32E-05	6.01E-06
	Cytokine activity	1.32E-05	6.01E-06
**KEGG pathway**	Bladder cancer	1.73E-07	1.04E-07
	Proteoglycans in cancer	2.05E-07	1.24E-07
	AGE-RAGE signaling pathway in diabetic complications	1.36E-05	8.24E-06
	Rheumatoid arthritis	1.90E-04	1.15E-04
	Viral protein interaction with cytokine and cytokine receptor	2.17E-04	1.31E-04

### Screening of Prognosis-Related OS Genes and Construction of a Genetic Risk Score Model for Patients With PC

**FIGURE 6:**
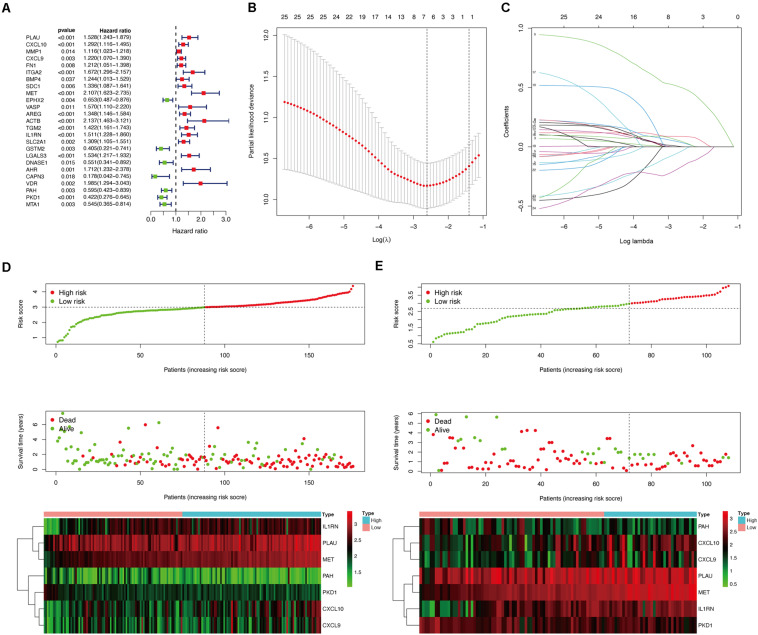
Construction of prognostic model in the TCGA and validation cohorts. **(A)** Univariate Cox regression analysis for identifying prognosis-related OS genes in TCGA cohort. **(B,C)** LASSO analysis for determining the number of factors and constructing the prognosis prediction model. **(D)** Risk score distribution, survival status, and expression heat map of TCGA cohort. **(E)** Risk score distribution, survival status, and expression heat map of validation cohort.

To further identify prognosis-associated OS genes, the 131 DEOGs identified from the PPI network were further analyzed using univariate Cox regression analysis, revealing 25 OS genes demonstrating significant (*P* < 0.05) associations with patient overall survival (Figure 6A). Thereafter, a LASSO algorithm was employed for specific OS gene range shrinkage (Figures 6B,C), and seven hub OS genes (PLAU, CXCL10, CXCL9, MET, IL1RN, PAH, and PKD1) were ultimately selected to compute the risk score. All PC patients in the TCGA (Figure 6D) or validation (Figure 6E) cohorts were separated into low- and high-risk subgroups according to the median risk score. The coefficients of the seven hub genes are shown in Table 2.

**TABLE 2:** Seven prognosis-associated OS genes with PC in the TCGA dataset were identified by LASSO analysis.

**OS name**	**Univariate Cox regression analysis**				**LASSO coefficient**	**Value of log lambda**
	**HR**	**Lower 95% CI**	**Upper 95% CI**	***P*-value**		
PLAU	1.5283	1.2431	1.8790	0.0001	0.0077	−2.6226
CXCL10	1.2917	1.1164	1.4947	0.0006	0.0879	−1.7799
CXCL9	1.2196	1.0701	1.3900	0.0029	0.0477	−2.1978
MET	2.1067	1.6230	2.7345	2.1552	0.5167	−1.1219
IL1RN	1.5113	1.2278	1.8602	0.0001	0.0620	−2.1525
PAH	0.5952	0.4225	0.8386	0.0030	−0.0620	−2.3475
PKD1	0.4222	0.2762	0.6454	0.0001	−0.1978	−1.6825

### Associations Between Prognostic Risk Score and Clinical Characteristics of PC Patients

**FIGURE 7:**
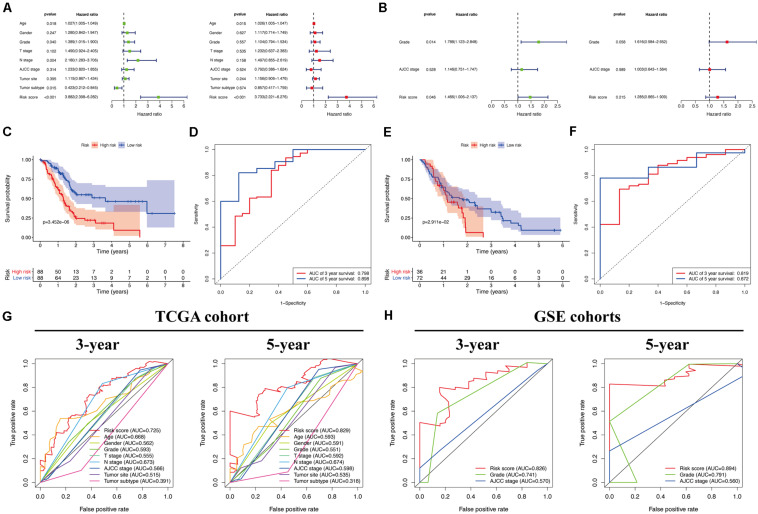
Efficacy evaluation of constructed prognostic model. Univariate and multivariate Cox regression analysis of the clinicopathological features in TCGA **(A)** and validation **(B)** cohorts. **(C)** Survival curve of TCGA cohort. **(D)** TimeROC curves for forecasting overall survival in TCGA cohort. **(E)** Survival curve of validation cohort. **(F)** TimeROC curves for forecasting overall survival in validation cohort. ClinicalROC curves for forecasting overall survival in TCGA **(G)** and validation **(H)** cohort.

Univariate and multivariate Cox regression analyses (Figures 7A,B) showed that our identified risk score was significantly connected with PC patient prognosis and emerged as an independent prognostic feature. Expectedly, the predictive value analysis of our risk score model in the TCGA cohort showed that it was significantly associated with the overall survival of patients with PC (*P* < 0.05), and the AUC (area under the receiver operating characteristic curve) reached 0.798 and 0.898 for 3- and 5-year survival, respectively (Figures 7C,D). Of note, the same prognostic capacity of seven genes’ prognostic signature was also validated in the GEO validation cohort. The survival analysis results also indicated that the overall survival of patients with PC was significantly decreased, as evidenced by an increased risk score in the validation cohort (*P* = 0.029; Figure 7E). In addition, time-dependent receiver operating characteristic (ROC) curve analysis of overall survival in patients with PC indicated that our prediction model had moderate predictive accuracy with an AUC value of 0.819 and 0.872 for 3- and 5-year survival, respectively, in the GEO cohorts (Figure 7F), which demonstrated that our prognostic model had reliable specificity and sensitivity for patients with PC. Moreover, while compared with other clinicopathological characteristics in the validation cohort, our ROC curve analysis indicated that our risk model outcompeted other diagnostic features in terms of reliably and accurately predicting 3- and 5-year survival (Figure 7H). Of course, this improved predictive value was also calculated in the TCGA cohort at 3 and 5 years (Figure 7G).

**FIGURE 8:**
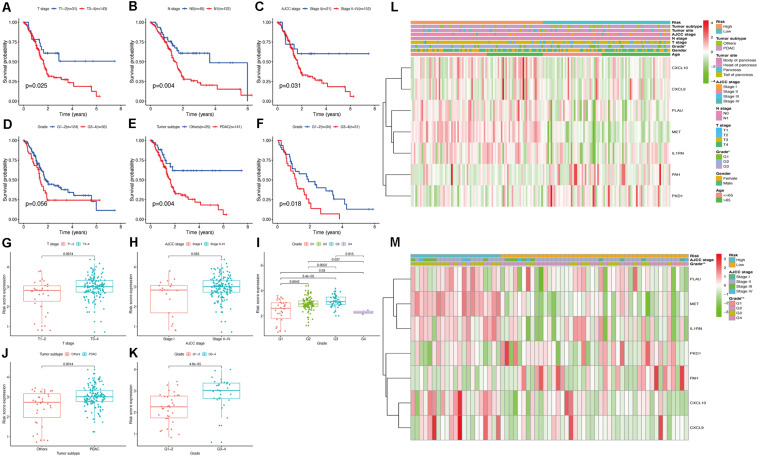
Evaluation the relationship between the risk score and clinicopathological parameters in patients with PC. **(A–E)** Survival curve of clinicopathological characters in TCGA cohort. **(F)** Survival curve of clinicopathological characters in validation cohort. **(G–J)** Correlation analysis between the risk score and clinicopathological characters in TCGA cohort. **(K)** Correlation analysis between the risk score and clinicopathological characters in validation cohort. The heatmap shows the distribution of clinicopathological features and OS genes expression in two risk subgroups from the TCGA **(L)** and validation **(M)** cohorts.

Pancreatic cancer at a higher T stage (Figure 8G), American Joint Committee on Cancer (AJCC) stage (Figure 8H), or tumor grade (Figure 8I) had a significantly increased risk score (*P* < 0.05), indicating that our risk model reliably predicted PC progression. Interestingly, tumors of grade 4 had the lowest risk score, which may be due to the minimal number of grade 4 PC tissues in the analyzed sample. Cancers histologically diagnosed as pancreatic ductal adenocarcinoma (PDAC) were significantly associated with higher risk scores than other PC subtypes in the TCGA cohort (*P* < 0.05; Figure 8J). In the validation cohort, patients with a higher tumor grade also had a higher risk score (*P* < 0.05; Figure 8K).

To further clarify the modulation mechanism of the risk score in predicting the overall survival of patients with PC, we also determined the relationship between the clinicopathological features and overall survival. The results indicated significantly poorer outcomes for PC samples of the PDAC subtype or PC samples with higher T stage, N stage, AJCC stage, and tumor grade (Figures 8A–F), suggesting that our risk model is strongly associated with the overall survival of PC patients by accurately predicting cancer progression and subtypes. Heatmaps constructed using the TCGA and validation cohorts for the expression levels of the seven hub OS-related genes in the two risk subgroups (Figures 8L,M) and showed significant differences in tumor grade between groups, in both cohorts (*P* < 0.05). These results indicated that our prognostic model has remarkable potential for predicting PC outcomes and clinical features.

### Prognostic Value of Hub OS-Related Genes

**FIGURE 9:**
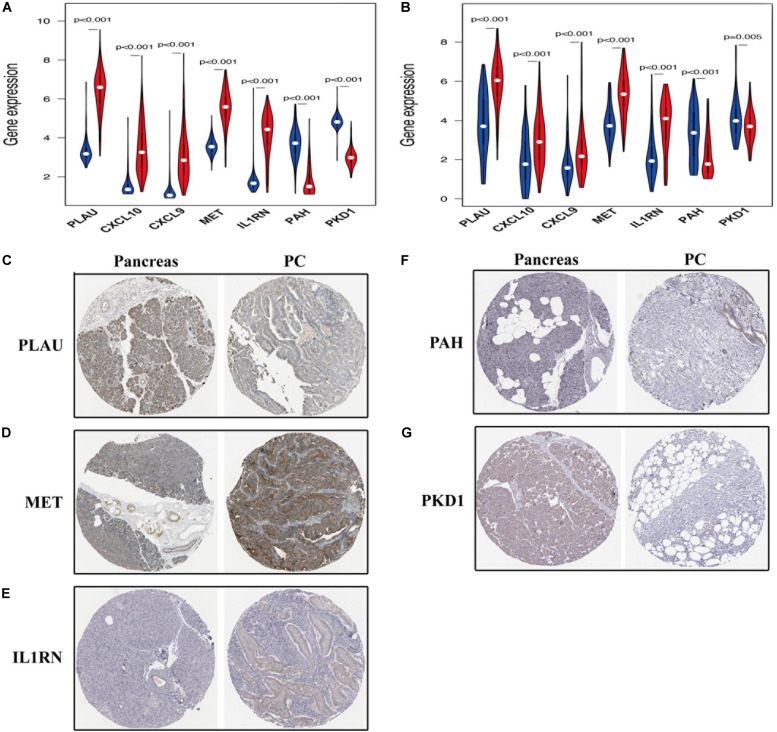
The expression of prognosis-related OS genes in patients with PC. The violin plot reveals the transcription expression of OS genes in TCGA **(A)** and validation **(B)** cohorts. HPA database verifies the protein expression of PLAU **(C)**, MET **(D)**, IL1RN **(E)**, PAH **(F)**, and PKD1 **(G)** in PC.

**FIGURE 10:**
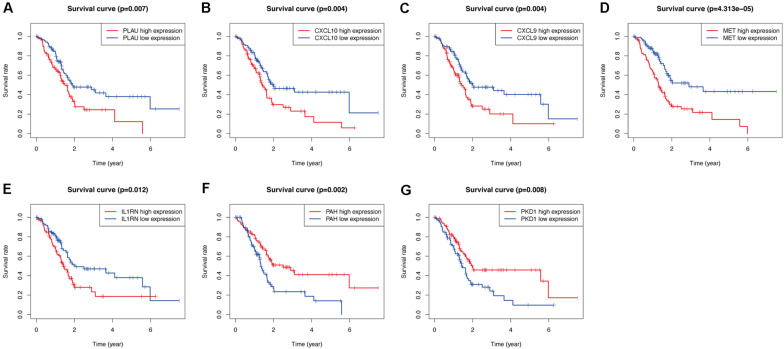
Validation the prognostic value of the prognosis-related OS genes of PLAU **(A)**, CXCL10 **(B)**, CXCL9 **(C)**, MET **(D)**, IL1RN **(E)**, PAH **(F)**, and PKD1 **(G)** in TCGA-PC cohort by Kaplan-Meier analysis.

As shown in Figures 9A,B, among the seven hub genes, the expression levels of PLAU, CXCL10, CXCL9, MET, and IL1RN were significantly elevated, whereas the expression levels of PAH and PKD1 were significantly decreased in PC samples compared with those in the normal pancreas samples. Similar results were obtained by analyzing these hub OS-related genes’ protein expression levels using the immunohistochemistry results from the HPA database (Figures 9C–G). Kaplan-Meier analysis further showed that the overall survival of patients with PC was inversely associated with the gene expression levels of PLAU, CXCL10, CXCL9, MET, and IL1RN (*P* < 0.05; Figures 10A–E); however, the expression levels of PAH and PKD1 had positive associations with the prognosis of patients with PC (*P* < 0.05; Figures 10F,G). A similar prognostic trend was also discovered in the validation cohort (Supplementary Figure 1), whereas only genes PLAU and MET genes were significantly associated with the prognosis of patients with PC (*P* < 0.05), which might be due to the small number of PC samples and the unequal composition of patients with PC (in the validation cohort, no PC patient had an overall survival of more than 3 years). Therefore, further experiments are warranted to validate the specific role of these seven hub OS-related genes in the prognosis of patients with PC.

### Nomogram Construction

**FIGURE 11:**
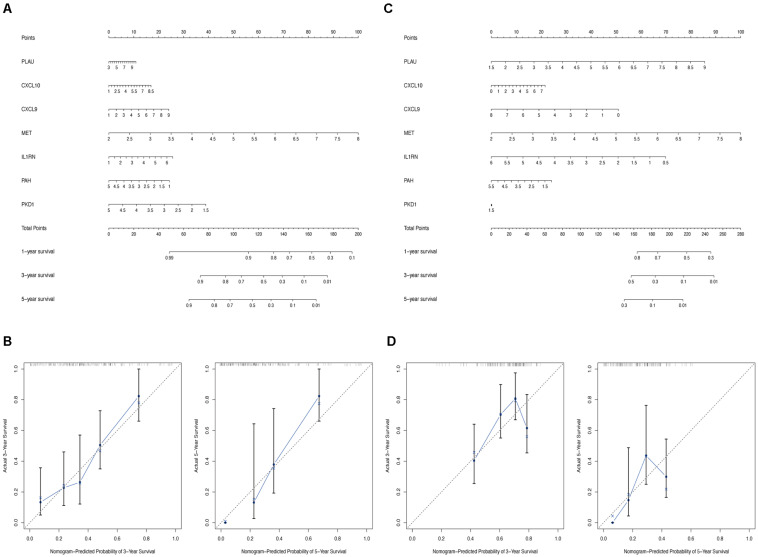
Construction of nomogram based on the expression of 7 hub OS genes. The nomogram **(A)** and calibration plots **(B)** of hub OS genes in TCGA cohort. The nomogram **(C)** and calibration plots **(D)** of hub OS genes in validation cohort.

Finally, to enable the identified hub genes to be applied for predicting the overall survival of patients with PC in a practical setting, the nomogram plots based on the expression levels of the seven hub genes were constructed to predict the clinical outcome of patients with PC in the TCGA-PC (Figure 11A) and validation cohorts (Figure 11C). The calibration plots demonstrated that our nomograms showed good agreement between the predicted and observed outcomes (Figures 11B,D).

## Discussion

Pancreatic cancer is one of the most common malignancies and a major cause of cancer-related deaths worldwide (Kamisawa et al., 2016). Although many novel diagnostic techniques and molecular biomarkers have been recently discovered, they have not sufficiently improved the early diagnosis and prognosis of patients with PC (Yan et al., 2019). Therefore, it is imperative to identify more PC prognosis-associated biomarkers and elucidate the precise mechanism underlying cancer progression. In the present study, we aimed to identify reliable molecular biomarkers for the prognostic assessment of PC and provide a basis for treatment decisions. To this end, we focused on OS as a confirmed mechanism of cancer progression and applied differential expression analysis to identify candidate DEOGs between PC and healthy pancreatic samples. A total of 148 DEOGs were selected for further exploration. In addition, the KEGG pathway enrichment analysis indicated that our identified DEOGs were not only significantly associated with the prognosis of pancreatic cancer, but also played a critical role in numerous other tumors, including bladder cancer, hepatocellular carcinoma, prostate cancer, melanoma, and colorectal cancer, prompting us to further explore the potential role of OS genes in other tumors.

The PPI network, univariate Cox regression, and LASSO analysis of the DEOGs identified a total of seven genes (PLAU, CXCL10, CXCL9, MET, IL1RN, PAH, and PKD1) as hub prognosis-associated genes for further exploration. The mRNA and protein expression profiles of these seven genes using the expression data from TCGA-PC and GEO (GSE28735 and GSE62452) cohorts and the HPA database revealed that PLAU, CXCL10, CXCL9, MET, and IL1RN were overexpressed, whereas PAH and PKD1 were downregulated in PC tissues. Kaplan-Meier analysis further revealed that these overexpressed hub genes were negatively associated with the overall survival of patients with PC, whereas PAH and PKD1 expression levels positively correlated with patient outcomes. These results might correspond with the modulation effects of these genes in PC metastasis and growth, as previously reported.

PLAU is reportedly significantly overexpressed in PC samples and associated with pancreatic cell invasive ability (Bournet et al., 2012; Liu et al., 2016). Several bioinformatics analyses also indicated that PLAU has prognostic value in PC (Lu et al., 2018; Chen et al., 2019). ELR-negative CXC chemokines, CXCL9 and CXCL10 were shown to induce lymphocytic migration and attenuate angiogenesis, leading to longer overall survival in patients with advanced PDAC (Qian et al., 2019). However, some studies also indicated that these chemokines might play tumorigenic roles by promoting tumor metastasis and proliferation (Mir et al., 2015; Wightman et al., 2015); thus, the specific roles of CXCL9 and CXCL10 in PC remain unclear. MET was originally identified as an oncogene that displayed 7-fold increased expression levels in PC samples, and its overexpression directly correlated with tumor grade and an aggressive PC phenotype (Modica et al., 2018). Protein kinase D1 (PKD1) is one of three members of the PKD family of serine/threonine kinases, which can be activated by intracellular OS (Waldron and Rozengurt, 2000), and its activation has proven to contribute to the initiation of PC (Döppler and Storz, 2017). Although some of our identified hub genes were previously reported to be significantly associated with PC progression, no study has systematically analyzed the specific prognostic role of OS genes in PC. In the current study, we demonstrated that the differential expression of seven hub OS-related genes is significantly associated with patients’ overall survival and involved in PC development. Nevertheless, to validate these OS-related genes as potential prognostic biomarkers for PC, more experimental evidences from prospective clinical and pre-clinical studies are needed. Future studies must verify whether PC patients could benefit from the modulation of these genes and the exact relationship between these genes and PC cells.

Moreover, to identify whether these specific OS genes could be used as prognostic factors, we constructed a novel prognostic prediction model based on the expression of the seven hub genes. To our knowledge, this is the first OS-associated risk model for prognostic prediction. Univariate and multivariate Cox regression analyses revealed that our risk model had reliable prognostic value for PC and could be used as an independent prognostic factor in PC. Survival and ROC analyses confirmed the advantage of the biological implications of our OS hub genes-related risk model for predicting PC prognosis. They showed improved predictive accuracy compared with conventional clinicopathological features, such as age, sex, AJCC stage, tumor grade, tumor site and tumor subtype. In addition, considering the critical role of OS in various stages of cancer progression and carcinogenesis (Reuter et al., 2010; Hecht et al., 2016), we further assessed the connections between risk score and PC clinical factors and discovered that the constructed risk model was significantly associated with T stage, AJCC stage, grade, and subtype of cancer samples, which was consistent with the prognostic effects of clinical features in overall survival. The AJCC staging system is one of the most widely used clinicopathological parameters for PC prognosis prediction (Kamarajah et al., 2017). However, the AJCC staging model is still not suitable for elucidating comprehensive PC behaviors and does not have sufficient diagnostic accuracy for PC (Yan et al., 2019). A similar conclusion was made in this study. Compared with the AJCC stage, our risk model not only showed a stronger relationship with PC prognosis but also could effectively predicted other PC features, including tumor grade and subtypes. These results indicate that our risk model has great advantages in the prognosis prediction of patients with PC. Our nomogram analysis confirmed the credibility of the identified OS genes in predicting the overall survival of patients with PC. Taken together, our results demonstrate the prognostic value of an OS-related gene-based risk model for patients with PC and suggest a novel method for evaluation the survival rate of PC patients.

Nonetheless, this study has some limitations. First, this study was designed as a retrospective analysis; thus, more prospective research should be performed to verify our results. Second, our results lack *in vitro* or *in vivo* exploration to confirm the reliability of the mechanism analysis. Therefore, several experiments are needed to prove the mechanistic connections between the identified hub genes and PC progression.

## Conclusion

In conclusion, through a series of bioinformatics analyses, we identified seven hub OS-related genes that are significantly associated with the overall survival of patients with PC. We also successfully established a prognostic model with powerful predictive effects and developed an effective nomogram composed of the gene signature in PC patients. Thus, our study foretells that these OS genes will greatly contribute to explain the pathogenesis and progression mechanism of PC and may serve as potential therapeutic targets to treat PC patients.

## Data Availability Statement

The original contributions presented in the study are included in the article/Supplementary Material, further inquiries can be directed to the corresponding author/s.

## Author Contributions

H-XJ and S-YQ conceived and designed the research, conducted the experiments, analyzed the data, and wrote the manuscript. XQ and Q-HH participated in the collection of clinical samples. Q-YS participated in the experimental design and provided financial and instrumental support. All authors read and approved the final manuscript.

## Conflict of Interest

The authors declare that the research was conducted in the absence of any commercial or financial relationships that could be construed as a potential conflict of interest.

## Footnotes

1 https://portal.gdc.cancer.gov

2 https://gtexportal.org/home/datasets

3 https://www.ncbi.nlm.nih.gov/geo/

4 https://www.genecards.org

5 http://www.string-db.org/

6 http://www.proteinatlas.org/

## Supplementary Material

Supplementary Figure 1 Validation the prognostic value of 7 prognosis-related OS genes in validation cohort by Kaplan-Meier analysis.

Supplementary Table 1 Details of datasets in this study.
