# Supplementary figures and images for: Identification of Hub Prognosis-Associated Oxidative Stress Genes in Pancreatic Cancer Using Integrated Bioinformatics Analysis
Source: Front Genet. 2020 Dec 8;11:595361. doi: 10.3389/fgene.2020.595361 (PMC7753072; doi:10.3389/fgene.2020.595361)

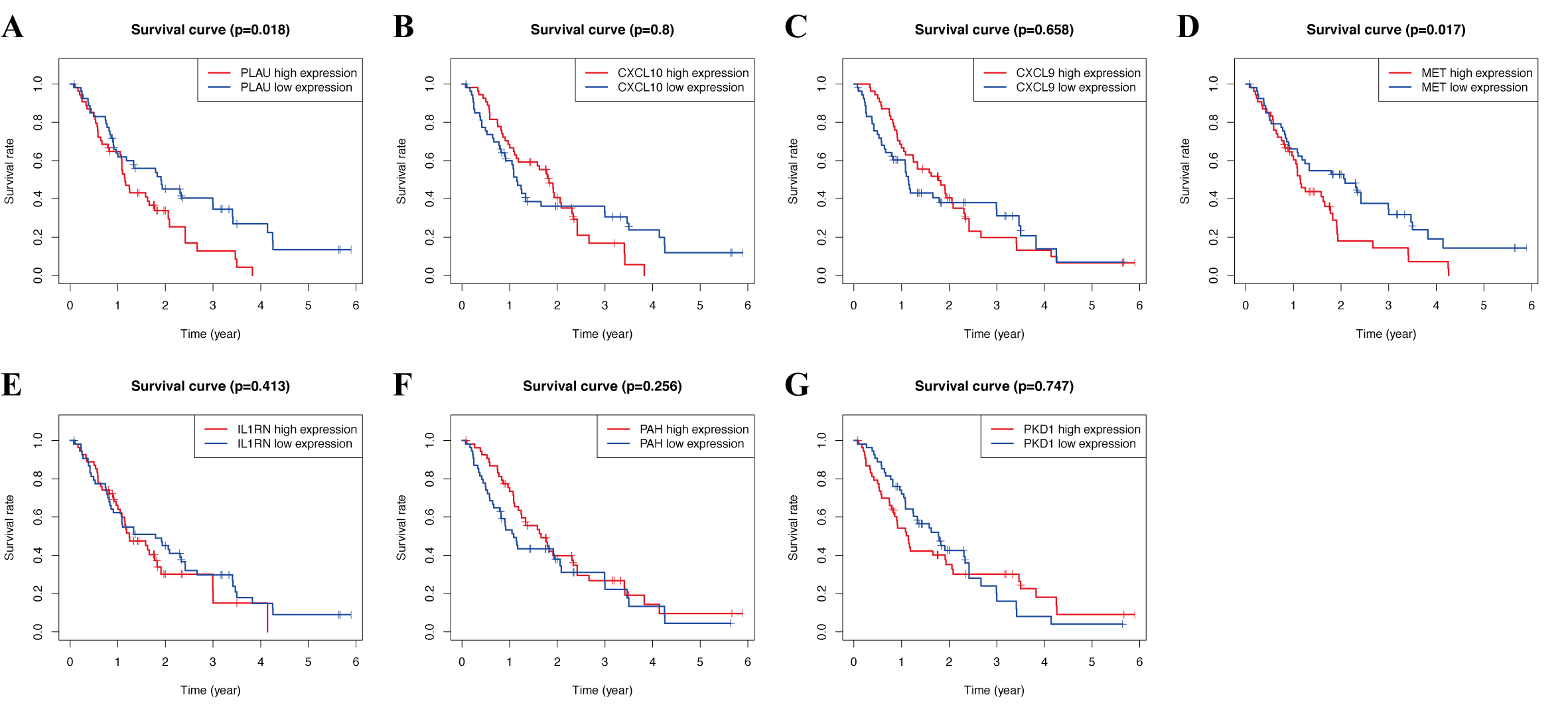

Supplement: Supplementary Figure 1 — Validation the prognostic value of 7 prognosis-related OS genes in validation cohort by Kaplan-Meier analysis. [file Image_1.TIF]
