# Supplementary material for: Identification of Hub Prognosis-Associated Oxidative Stress Genes in Pancreatic Cancer Using Integrated Bioinformatics Analysis
Source: Front Genet. 2020 Dec 8;11:595361. doi: 10.3389/fgene.2020.595361 (PMC7753072; doi:10.3389/fgene.2020.595361)
Supplement: Supplementary Table 1 — Details of datasets in this study. [file Table_1.docx]

**SUPPLEMENTARY TABLE 1 |** Details of datasets in this study.

| Accession number | Platform | Number of samples | | Country | Version |
| --- | --- | --- | --- | --- | --- |
|  |  | Non-tumor pancreatic tissue | pancreatic cancer |  |  |
| TCGA | IlluminaHiSeq | 4 | 178 | USA | 2019 |
| GTEx | / | 167 | / | / | 2016 |
| GSE28735 | Affymetrix | 45 | 45 | USA | 2018 |
| GSE62452 | Affymetrix | 61 | 69 | USA | 2018 |
| Total |  | 237 | 292 |  |  |
